# Complete Genome Sequence of an NADC30-Like Strain of Porcine Reproductive and Respiratory Syndrome Virus in China

**DOI:** 10.1128/genomeA.00303-16

**Published:** 2016-04-21

**Authors:** Guobiao Ji, Yingying Li, Feifei Tan, Jinshan Zhuang, Xiangdong Li, Kegong Tian

**Affiliations:** a College of Animal Science and Veterinary Medicine, Henan Agricultural University, Zhengzhou, China; b National Research Center for Veterinary Medicine, High-Tech District, Luoyang, China; c Veterinary Diagnosis Center and OIE Porcine Reproductive and Respiratory Syndrome Laboratory, Beijing, China

## Abstract

The most recently emergent porcine reproductive and respiratory syndrome virus strains in China are characterized by 393 nucleotide deletions in the nonstructural protein 2 (NSP2) region and are known as NADC30-like strains. Here, we report the complete genome sequence of the NADC30-like HNjz15 strain that was isolated in 2015.

## GENOME ANNOUNCEMENT

Porcine reproductive and respiratory syndrome virus (PRRSV) is a major swine pathogen causing huge economic losses in the swine industry worldwide. PRRSV belongs to the order *Nidovirales*, family *Arteriviridae*, and can be divided into European genotype 1 and North American genotype 2. PRRSV has shown remarkable genetic variation through mutation and recombination, resulting in new isolates with different levels of pathogenicity and virulence. Highly pathogenic PRRSV (HP-PRRSV) with a discontinuous 30-amino acid (aa) deletion has been circulating and predominating in the field since 2006 and has affected >2 million pigs in China (1, 2). Recently, several field isolates of PRRSV that are genetically similar to the NADC30 strain, a type 2 PRRSV that was isolated in United States in 2008, were designated NADC30-like PRRSV and caused the outbreaks of severe PRRS (3–5). Here, we report the complete genome sequence of the NADC30-like variant HNjz15.

The HNjz15 strain was isolated in 2015 from the serum samples of diseased pigs using porcine pulmonary alveolar macrophages. The first passage of virus was used for genomic sequencing. Basically, 12 primer pairs were used to generate overlapping amplicons by reverse transcription-PCR (RT-PCR). The 5′ and 3′ regions were obtained by rapid amplification of cDNA ends (RACE) using a RACE kit (TaKaRa, China). The PCR products were cloned into pEASY-Blunt vector (TansGen Biotech, China), sequenced three times, and assembled into the full-length sequence using the DNAStar (Lasergene) software. The genome of HNjz15 was 15,019 nucleotides in length, excluding the poly(A) tail.

Genome analysis results showed that HNjz15 shared the highest similarity with NADC30 (95.6%) compared to other PRRSV strains. Like other NADC30-like isolates, HNjz15 had the same discontinuous amino acid deletions in the nonstructural protein 2 (NSP2) region as those in NADC30 (GenBank accession no. JN654459). These deletions were identified as 1 aa at position 303, 111 aa at positions 323 to 433, and 19 aa at positions 501 to 519. In addition, it is noteworthy that HNjz15 had 2 amino acid mutations at sites 12447 (G to A) and 13067 (A to G), which led to an early termination of the GP2 protein code and a delayed termination of the GP3 protein code. Consequently, the number of amino acids of the GP2 protein of HNjz15 was 1 amino acid less than that of NADC30, and the number of amino acids of GP3 protein was 12 amino acids more than that of NADC30. The significance of these amino acid changes on the pathogenicity of NADC30 is worth further investigation.

The genome data of HNjz15 will help us to understand the epidemiology and evolution of PRRSV in pigs.

### Nucleotide sequence accession number.

The complete genome sequence of the HNjz15 strain is available in GenBank under the accession no. KT945017.
